# Good News or Bad News? How Message Framing Influences Consumers’ Willingness to Buy Green Products

**DOI:** 10.3389/fpsyg.2020.568586

**Published:** 2021-01-19

**Authors:** Zelin Tong, Diyi Liu, Fang Ma, Xiaobing Xu

**Affiliations:** School of Management, Hainan University, Haikou, China

**Keywords:** psychological distance, framing effect, willingness to buy green products, emotional mechanisms, spatial distance

## Abstract

Despite the growing social interest in green products, companies often find it difficult to find effective strategies to induce consumers to purchase green products or engage in other environmentally friendly behaviors. To address this situation, we examined the favorable or unfavorable effects of positive and negative message frames on consumers’ willingness to consume green products in different psychological distance contexts. Through two Studies, we found that the positive information framework played a more pronounced role in context when consumers were in closer spatial distances. More importantly, we found that the emotional factors of fear and hope were intrinsic causes of this phenomenon. Correspondingly, the negative information framework played a better facilitating role in context with farther spatial distance, while shame and pride were the emotions responsible for this effect. Finally, we discuss the theoretical and managerial implications of our work, as well as its limitations and future research directions.

## Introduction

Green products are those that are manufactured with care to minimize the exploitation of natural resources, use of toxic materials, or emissions of waste and pollutants (Lin and Chang, 2012; Haws et al., 2014). The data indicate that companies across industries are increasingly interested in producing and selling environmentally sustainable products (Delmas and Burbano, 2011; Romani et al., 2016). However, it is often difficult for companies to design communication strategies that increase consumers’ willingness to choose these products (Kalamas et al., 2014). For this reason, one strategy often employed by firms is the use of positive or negative framing (Tversky and Kahneman, 1981; Levin et al., 1998). Through positive framing messages, firms highlight the potential environmental benefits of purchasing green products. Conversely, through positive framing messages, companies emphasize the harmful environmental consequences of consumers not purchasing sustainable products. The questions are which framing messages are better for consumers, and how different framing effects influence the consumers’ willingness to buy green.

Existing green product communication research diverges on the role of different framing messages, with some studies arguing that negative information frameworks may be more effective than positive ones in promoting green procurement and other responsible behaviors (e.g., Olsen et al., 2014). Others disagree, for example, as some studies suggest that positive frames may be more effective when it comes to promoting risk aversion/prevention behaviors (Dijkstra et al., 2011), while in many cases, environmental protection can be seen as a way to encourage people to take steps to prevent environmental damage (Loroz, 2007), and thus is likely to yield different results. Previous research has shown that the most immediate effect of different framing messages on people is not a long-term cognitive one (Such as alertness, severity judgments, etc.), but rather an immediate emotion that is triggered (McElroy and Seta, 2004). This emotion, while not necessarily long-lasting and sometimes hidden (Sun et al., 2020), has an immediate and more significant impact on consumers’ willingness to purchase. We argue that different framing messages are used to influence consumers’ green purchase intentions by affecting people’s emotions, an effect that is far more pronounced than the cognitive pathway. Of course, the framing effect on people’s emotions is not invariant; positive or negative framing affects people’s emotions differently when the psychological distance, especially in the spatial dimension, varies. Specifically, we argue that when the spatial distance is close, negative information framing tends to bring fear and cause avoidance behavior, which in turn reduces consumers’ willingness to make green purchases, whereas positive frames are motivated by creating a sense of hope, which in turn increases consumers’ willingness to buy green. At the same time, in the case of long spatial distances, a negative message frame triggers a sense of shame and thus achieves better results than a positive message frame.

This research makes two major theoretical contributions. First, we argue that the psychological distance variable plays a significant moderating role in studies of framing effects on people’s prosocial behavior (White et al., 2011); specifically, the effect of negative framing on people should not be favored when psychological distance is sufficiently close. Second, we extend the psychological distance-related theory from another perspective, that is, in addition to its significant impact on people’s explanatory level cognition and thus on their behavior (Levin et al., 2002), the impact of psychological distance on people’s moods and emotions should also receive attention. For example, people’s attention to environmental issues tends to differ across spatial dimensions, and our Studies pave the way for an underlying mechanism that the phenomenon is most likely related to the different emotions triggered by psychological distance.

## Theoretical Background and Assumptions

The framing effect refers to the phenomenon in which a change in the way an option is described leads to a reversal of an individual’s choice preferences. Tversky and Kahneman (1981) called this phenomenon a “framing effect.” De Martino et al. (2006), on the other hand, argue that the “framing effect” evokes an emotional message, an emotional heuristic.

The application of framing effects to social behavior, particularly green behavior, is not uncommon, but its utility has been debated. For example, some studies have argued that negative framing messages stimulate protective mechanisms and thus have a more significant effect on pro-social behavior (Arthur and Quester, 2004). Other studies have argued that negative frames do not work better in all contexts, such as White et al. (2011) study, which showed that positive frames work better when the information given was at a high level of interpretation, or abstract, while negative frames worked better when the information given was at a low level of interpretation, or concrete. Other studies, however, have argued that positive frames give a higher perceived value and thus have better results in environmental protection behavior (Liu and Gu, 2020). Although it is true that alertness has an impact on people’s environmental behavior (Sun et al., 2018; She et al., 2019), and some studies have attempted to explain the role of the framing effect in terms of level of explanation (which affects alertness and perceptions of severity) (White et al., 2011), research has shown that the framing effect only works when activated in the right half of the human brain (which primarily controls emotions and abstract perceptions) (McElroy and Seta, 2004). That is, the effects of the framing effect are not achieved through detailed processing of information (e.g., judging the severity of an event), but rather are realized through abstract perceptions such as emotions.

In order to further investigate the theoretical mechanisms responsible for this phenomenon, we first start with theories related to emotions and determine which emotions are mainly triggered by the framing effect in environmental activities. Emotion appraisal theory describes in detail the conditions under which 17 emotions arise by distinguishing four dimensions: the causality of the event, the degree of controllability, goal congruence (whether it yields positive or negative emotions), and the degree of arousal. For example, when people are confronted with an uncertain event caused by an uncontrollable cause and degree of uncertainty, if the event is inconsistent with their goals, they will primarily produce negative emotions of fear, whereas if the event is consistent with their goals, they will primarily produce positive emotions of hope. When people are confronted with an uncertain event caused by themselves, if the event is consistent with their goals, they will primarily produce positive emotions of pride, whereas if the event is inconsistent with people’s goals, then they will primarily generate negative emotions of shame (Roseman, 1991, p. 193). Furthermore, the results of several studies have validated and added to this theory, particularly the idea that some emotions should also be related to the person the event affects (or is expected to affect). For example, people primarily generate emotions of hope when they believe that the event will have an expected positive effect on them and they have little control over that effect (Pekrun, 2006), and they have emotions of pride when they realize that their actions have achieved some good outcome or had a good effect on the outside world. Previous research has also shown that an important distinction between fear and shame/guilt is whether the event will have dire consequences (or punishment) for themselves (Freud, 1923/1961; Ausubel, 1955; Kemper, 1978; Higgins, 1987). To summarize, when people are confronted with an event that has an indeterminate degree of consequence, emotions of hope arise if the event will affect them and the event is consistent with their goals, emotions of fear arise if the event is not consistent with their goals, emotions of pride arise if the event does not affect them and the event is consistent with their goals, and emotions of shame arise if the event is not consistent with their goals. And these contexts correspond to the framing effect in environmental issues.

The classical framing effect is to elicit positive/negative evaluations by describing the gains and losses of an event, while positive framing is to describe good and desirable goals, and negative framing is to describe unwanted situations (De Martino et al., 2006), in terms of goal congruence characteristics with significant differences. Since people, when getting information about environmental issues, default to the fact that the matter is not completely controllable (even if you tell them about the possible consequences of the event) (Leiserowitz et al., 2013), environmental protection situations are events that are not completely certain for the participant. At this point, based on previous inferences, when the spatial distance is close because of the impact that the environmental issue and the outcome of the environmental protection measures will have on the people themselves, they will have feelings of hope if the environmental issues they face are consistent with their goals, and feelings of fear if the environmental issues they face are not consistent with their goals. When events occur at a distance, since the governance of environmental issues is initiated by themselves and will change the situation of others, emotions of pride arise when the environmental issues are aligned with their goals, and emotions of shame arise if the environmental issues are not aligned with their goals. We therefore speculate that:

H1a: When the spatial distance is close, positive frames elicit emotions of hope, while negative frames elicit emotions of fear.
H1b: When the spatial distance is large, positive frames elicit feelings of pride, while negative frames elicit feelings of shame.

Afterward, we further investigate how these sentiments influence consumers’ green purchase intentions. The authors of previous research have argued that positive emotions are the good feelings that arise when an individual wants to smile when things are going well or the pleasure that arises when a stimulus satisfies a physiological need, contributes to the achievement of a personally relevant goal, or progresses smoothly (Cabanac, 1971; Carver, 2003). Lazarus states that hope may arise from unsatisfactory situations such as those that are damaging, threatening, or involving poverty. Snyder argues that hope may also originate in situations that are already satisfactory but can be improved. Both situations seem plausible. In terms of the utility of hope as a component of the meaning of people’s lives (Feldman and Snyder, 2005), it can help individuals establish goals that effectively overcome difficulties and find more ways to overcome them (Cheavens et al., 2006). Research on the sources of a sense of hope has also shown that hope can inspire a desire for a better situation and motivate people to work harder to achieve their goals. Therefore, we believe that eliciting feelings of hope in environmental issues can be instrumental in motivating people’s environmental behaviors and evoking environmental awareness.

Pride has long been an important research topic in the field of social psychology, and Weiner (1985) earlier defined pride as a positive, self-conscious emotion that people feel because they have taken on social responsibility and brought about positive outcomes. Authentic pride is associated with a sense of accomplishment and refers to the pride that results from an individual’s unstable and controlled internal attribution of success (“I did well because I personally tried”), which fosters empathy for an external group and helps people develop genuine, deep self-esteem (Williams and DeSteno, 2009; Ashton-James and Tracy, 2012). Pride plays a number of roles in marketing, for example pride has a negative effect on mass consumption (Han et al., 2007), however, this has some conflict in calling for the use of products with uniformly ‘green’ characteristics. In addition, pride increases consumers’ self-awareness and makes them prefer practical goods (Wilcox et al., 2010), however, the price of green products is generally high, so pride has limited effect on consumers’ green product intention.

Negative emotions are the basic subjective experience of an individual feeling down and trapped in an unpleasant situation and include a variety of distasteful emotions such as anger, shame, hatred, negative illness, fear, and tension (Watson et al., 1988). Negative emotions have been widely used in the social sciences and have received widespread attention in pro-social behavior, especially in environmental protection. For example, fear and shame are often associated with research on green purchasing behavior, and Witte (1992) argued that fear messages trigger fear control and danger control messages. O’Neill and Nicholson-Cole (2009) research suggests that the role of fear appeals is unstable across contexts, and that environmental protection behavior, as a typically precautionary motivated behavior, is a function of many of the fear appeals (Witte, 1992). The positive effect is not necessarily that the fear emotion plays a role but that the appeals message itself carries a certain preventive message (Ruiter et al., 2001). Conversely, for the emotion itself, fear may inhibit the establishment of preventive motivation by stimulating fear control processes and can create a desire to escape, even to flee immediately to a safe place (Roseman et al., 1994; McDonald et al., 2015). Thus, eliciting fear in environmental behavior acts as a hindrance to stimulating people’s environmental awareness. Summing up the above narrative, we hypothesize that:

H2a: When spatial distance is close, positive information induces feelings of hope and helps to increase the willingness to purchase green products.
H2b: When the spatial distance is close, negative information induces fear and impedes increasing the willingness to buy green products.

Another very important emotion in environmental behavior is shame, which Niedenthal et al. (1994) identified as an anxiety generated by negative self-evaluation; Patrick et al. (2009) also studied that, in addition to feeling shame for something they have already done, people can also experience this emotion by simply considering actions that may have effects on themselves, even if the actions have not yet occurred, thus creating the expected sense of shame when people imagine that they may have committed crimes. Past research has shown that people respond to shame in two important ways: by withdrawing from any action and thus avoiding further threats to their self-concept (Tangney and Dearing, 2002), or by taking actions that may restore their self-concept, such as environmental behaviors. Existing research (De Hooge et al., 2008, 2010, 2011) suggests that the tendency of individuals to engage or withdraw from environmental issues depends largely on whether the social environment offers opportunities to restore particular aspects of the “self” that are threatened. Consistent with this reasoning, we argue that if consumers are motivated to feel shame (e.g., by purchasing an environmentally unsustainable product), they will take advantage of any opportunities offered to them in their environment (e.g., by choosing an environmentally friendly product or by submitting to an environmental association, choosing an environmentally friendly product or making a donation to an environmental association) to avoid the threat of shame. We therefore argue that eliciting shame in environmental issues helps promote environmental behavior. Summing up the above narrative, we hypothesize that

H3: When spatially distant, negative frames induce feelings of shame and help increase the willingness to purchase green products.

In general, most of the existing theories explain the interaction of the framing effect with psychological distance from a cognitive perspective, and less from an emotional perspective. Therefore, this study aims to explore the mechanisms by which the framing effect brings about emotional changes in different psychological distance contexts, and thus triggers in different consumer behaviors.

## Study

### Study 1

Study 1 used an online questionnaire designed to test the main effect of the Study, that is, whether positive and negative message frames play different roles in the context of different psychological distance spatial dimensions. In this way, Study 1 provides preliminary evidence for the moderating role of the psychological distance spatial dimension in the effect of framing on green purchase intentions.

#### Method

We surveyed 421 respondents by means of an online questionnaire and randomly divided them into four groups (positive frame vs. negative frame, near vs. far spatial distance) according to spatial distance and framing effects. Respondents were first told that they would be participating in a behavioral research survey on a global issue, and then they were asked to read a text and imagine themselves in the scenario presented in the text: the scenario presented an environmental pollution problem, and two groups were told that the problem occurred in their city, while the other two groups were told that the problem occurred in a city downstream from them, but that the problems were all of their own making. They were then uniformly told that there was a new battery and that it was an environmentally friendly green product (the subjects were told about the battery’s function and applicability) (see in Appendix). However, given that sustainable products are typically more expensive than their conventional counterparts (Dale, 2008), the sustainable version of batteries is 20% more expensive than the less sustainable version (non-rechargeable) (see Van Doorn and Verhoef, 2011, for an investigation of consumer willingness to pay premium price for sustainable products). Two groups in the same scenario were told that using the battery would solve their environmental problems and the other group was told that if they did not use the battery, it would lead to an environmental collapse.

After reading the scenarios, the participants were instructed to complete a questionnaire about their willingness to purchase green products on the same screen. To ensure that the participants paid attention to the section describing spatial distance, questions were included to reinforce their focus on spatial distance (e.g., “I think this is close at hand”) and to provide an initial measure of the manipulation effect of the spatial distance variable, as measured by a 7-point Likert scale.

It is worth mentioning that the use of an online questionnaire allowed us to ensure that the selection of all respondents was completely random. In addition, we informed all respondents at the beginning of the survey that all their responses were recorded anonymously, meaning that the respondents knew that their responses could not be identified to the person who responded.

#### Result

Since spatial distance and framing effects were used as manipulated variables in the Study, we coded them as two bicategorical grouping variables [i.e., PD (psychological distance) = 1 when spatial distance is far and PD = 0 when spatial distance is close; Framing = 1 when the participant receives positive frames and Framing = 0 when negative frames]. Then, we performed a Two-way ANOVA on the obtained data and the results showed that the interaction effect of frame effect and spatial distance had a significant effect on the green product purchase willingness (*F* = 7.58, *p* < 0.01). Therefore, we further tested the moderating effect of spatial distance. Specifically, when the spatial distance was closer, the frame effect on the green product purchase indicated willingness. There was a significant positive effect, that is, the subjects’ green product purchase intention was higher in the positive information frame than in the negative information frame (*F* = 3.86, *p* < 0.05). In the spatially distant situation, consumers’ purchase intention was higher in the negative information frame (*F* = −3.61, *p* < 0.1).

**FIGURE 1:**
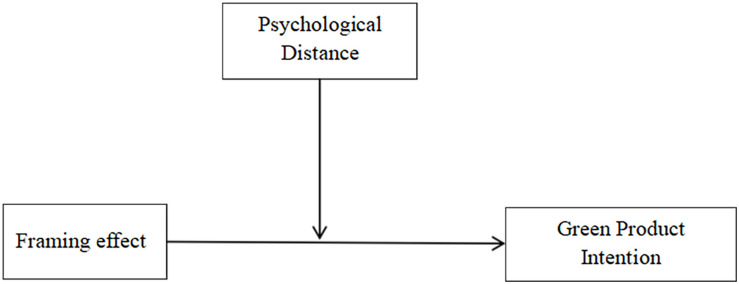
Model of Study 1.

In general, the results of Study 1 confirm our main effect conjecture that the positive frame is more effective than the negative frame in eliciting consumers’ willingness to buy green products in situations where the spatial distance is short, while the opposite is true in situations where the spatial distance is long (see in Figure 1). We will further investigate the underlying mechanisms in subsequent Studies.

### Study 2

Study 2 aimed to test the conjecture of H2 and H3 that, in the case of close spatial distance, negative information frames elicit respondents’ feelings of fear, which in turn adversely affects their green purchase intentions, whereas positive information frames elicit respondents’ feelings of hope, which in turn promotes their green purchase intentions. In the case of distant spatial distance, negative information frames elicit respondents’ feelings of shame, which in turn promotes their green purchase intentions.

#### Method

We also took the form of a web-based survey and randomly collected 202 samples (115 males, 87 females, MAge = 28.71, SD Age = 7.07) and 212 samples (105 men, 97 women, MAge = 28.77, SD Age = 8.50) in coastal urban areas (cities in Guangdong and Zhejiang) and non-coastal cities (e.g., Beijing, some cities in Shanxi). We further divided them into two groups (positive frame vs. negative frame) in each spatial distance context, depending on the frame effect that gave them information. As in Study 1, subjects were told that they were participating in a behavioral survey on global issues, that is, they were unaware of the specific purpose of taking part in the Study. They were then asked to watch a text and imagine themselves in the situation. This time, the situation indicated that their city (vs. the coastal city of their country) was facing a huge problem, namely sea level rise due to greenhouse gases, and the participants in the negative frame group were told that if none of them took action to reduce their own carbon emissions, it would lead to an increase in sea level rise (see in Appendix). The survey specified the devastating environmental impacts that come with rising sea levels. Participants in the positive frame group were told that if they all took positive action to reduce their own carbon emissions, they could significantly reduce the problems they faced, and at the same time, make the environment they lived in a better place.

After reading the contextual information, they were asked to fill out a questionnaire that asked about their fears (Hoelter, 1979), their shame (Han et al., 2014), pride (Tracy and Robins, 2006), and their hope (Snyder, 1995). In addition, before answering these emotion measurement questions, they were asked to investigate their views on the matter (e.g., I would wait and see what happens, etc.) to elicit their emotional factors.

Subsequently, the participants were told that a company had introduced a new environmentally friendly air conditioner with energy-saving features, and the participants in the positive frame group were then told that if they all purchased this energy-saving air conditioner, the sea-level rise problem they were facing would be greatly alleviated and their environment would be further improved. The subjects in the negative frame group were told that if they did not switch to this air conditioner, it would cause further problems and even lead to the collapse of their environment. They were then asked about their willingness to buy this product. All the items asked above were measured using a 7-point Likert scale.

As in Study 1, the participants in Study 2 were told to fill out the questionnaire anonymously, but the difference was that the subjects in Study 2 came from locations with different characteristics (i.e., coastal or non-coastal) and thus had different perceptions of the spatial distance of coastal problems, which could trigger different emotions.

#### Result

First, for ease of presentation, we combined the measures via factor scores into the variables “fear” (α = 0.80), “shame” (α = 0.84), “pride” (α = 0.77), and “hope” (α = 0.73). Subsequently, we performed ANOVA analysis on the emotions elicited by the frame effect in the spatially close and spatially distant groups, respectively, and the data showed that when spatially close, the frame effect had a significant effect on fear (*F* = 13.92, *p* < 0.01) and hope (*F* = 10.24, *p* < 0.01), whereas it had a non-significant effect on shame (*F* = 0.79, *p* > 0.1) and pride (*F* = 1.25, *p* > 0.1). When spatial distance distant, the frame effect was significant for shame (*F* = 5.48, *p* < 0.05) and pride (*F* = 8.73, *p* < 0.01), but not for fear (*F* = 0.108, *p* > 0.1) and hope (*F* = 1.56, *p* > 0.1). Thus validating our H1 conjecture.

**FIGURE 2:**
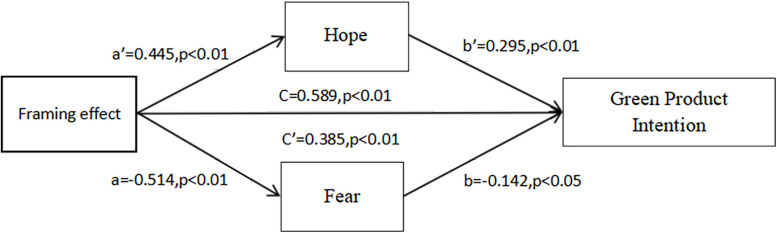
Model of PD = 0.

In the ANOVA test of the effect of frame on the willingness to buy, the results again confirmed the results of Study 1, that is, in the case of close spatial distance, the positive frame group was more willing to buy green products than the negative frame group (*F* = 18.7, *p* < 0.01). Subsequently, we used SPSS PROCESS Macro by Hayes (2013, model 4) to perform a mediation test between fear and hope emotions in Study 2. The results showed that the frame effect had a significant direct effect on purchase intentions (*B* = 0.385, *t* = 2.81, *p* < 0.01), while the frame effect had a significant negative effect on fear emotions (*B* = −0.514, *t* = −3.73, *p* < 0.01), and fear had a significant negative effect on consumers’ green purchase intentions (*B* = −0.142, *t* = −2.12, *p* < 0.05), while the frame effect had a significant indirect effect on purchase intentions through fear (*B* = 0.07, CI 0.01, 0.17); that is, fear mediated the effect of the frame effect on purchase intentions. The frame effect had a significant positive effect on hope emotion (*B* = 0.445, *t* = 3.20, *p* < 0.01) and hope emotion had a significant positive effect on consumers’ willingness to purchase green products (*B* = 0.295, *t* = 4.45, *p* < 0.01), while the frame effect was significant through the indirect effect of hope on purchase willingness (*B* = 0.13, CI [0.06, 0.23]); that is, hope and fear fully mediates the frame effect on purchase intentions (see in Figure 2). This result validates our H2 conjecture from the data.

**FIGURE 3:**
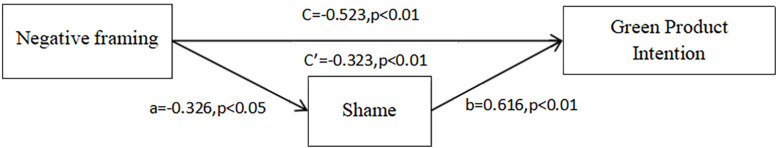
Model of PD = 1.

Subsequently, we tested the data of the two groups with far spatial distance. In the ANOVA test for the effect of the frame effect on purchase intentions, the results were opposite to Study 2, again providing data to support Study 1 that the negative frame was more likely to purchase green products than the positive frame situation (*F* = 13.54, *p* < 0.01). Subsequently, we used SPSS PROCESS Macro (Hayes, 2013, model 4) to conduct a mediation test of shame and pride in the spatially distant group, which showed a significant direct effect of framing on purchase intentions (*B* = −0.323, *t* = −2.93, *p* < 0.01 CI −0.54, −0.11). The frame effect has a significant negative effect on shame (*B* = −0.326, *t* = −2.34, *p* < 0.05), while shame has a significant positive effect on the willingness to purchase green products (*B* = 0.616, *t* = 11.18, *p* < 0.01), while the frame effect has a significant indirect effect on the willingness to purchase through shame (*B* = −0.20, CI −0.38, −0.04); that is, shame and pride mediated the frame effect on purchase intentions. However, the difference is that the effect of pride on purchase intention is not significant (*p* > 0.1, CI [−0.06, 0.16]), that is, pride does not significantly affect consumers’ willingness to purchase green products (see in Figure 3). This result validates our H3 conjecture in terms of data.

**FIGURE 4:**
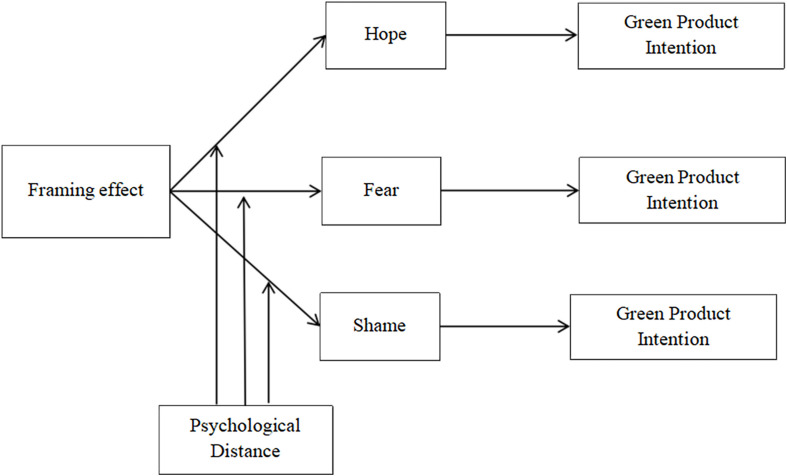
Model of full Study.

Overall, Study 2 verified the mediating role of emotions, especially fear, shame, and hope, on framing effects and purchase intentions, which explains the higher effect of positive framing on people’s green product purchase intentions relative to negative framing when spatial distance is close, and the better effect of negative framing on people’s green purchase intentions relative to positive framing when spatial distance is far (see in Figure 4).

## Discussion

As the problem of subsistence living fades away and people put forward higher demands on health and environment, an increasing number of companies are focusing on green products, and they want to find a way to increase consumers’ willingness to buy green products to increase their companies’ revenues. Although companies are now more sensitive to the topic of green products, they still have limited information on how to increase their green purchasing intentions. To date, there is still much room for development in the field of research on how to increase consumers’ willingness to buy green products, especially on how to interpret the influence of various messages on consumers’ green behavior from an emotional point of view. In this context, we designed two Studies to investigate the effects of different information framing effects on consumers’ green purchase intentions in the context of different spatial distances, and also to investigate the mediating role of emotions in this process.

In Study 1, we focus on exploring how the framing effect influences consumers’ willingness to purchase green products disparately in the context of different spatial distances. In line with our hypothesis, spatial distance significantly moderates the frame effect on consumers’ willingness to purchase green products. In previous studies, the effect of negative framing on social behavior has often been perceived as more pronounced because negative framing can often lead people to believe that they are making a more effective contribution (White et al., 2011). As previously mentioned, when bad things are close enough to you, people tend to try to avoid the problem because of emotions (especially fear). The results indicated that instead of people’s trying to overcome the problem, this is one of the effects of negative emotions on people’s behavior (Witte, 1992). The data we obtained support the above point.

In Study 2, we explored the mediating role of specific emotions in this influence based on the phenomenon of Study 1 and verified our H2 that in situations where the spatial distance is close, positive frames trigger hopeful emotions and therefore have a positive impact on purchase intentions, and negative frames trigger fears and thus weaken people’s willingness to buy green products. It was then further verified that H3, that is, in the context of further emotional distance, the positive frame would trigger people’s pride emotions and increase their environmental awareness, but have less influence on green product purchase behavior, while the negative frame would trigger people’s shame emotions and thus increase people’s willingness to purchase green products.

## Theoretical Implications

This study makes an important contribution to theoretical systems that believe that emotions play an important role in sustainable development (Moons and De Pelsmacker, 2012) by exploring the role of four emotions – fear, shame, pride and hope – triggered by different framing effects, combined with the partial theory of mental distance, in influencing people’s willingness to buy green products.

First, we argue that for studies of framing effects on people’s pro-social behavior (White et al., 2011), the variable of psychological distance plays a significant moderating role; specifically, the effects of negative framing on people should not actually be viewed favorably when the psychological distance is close enough.

Second, we extend the psychological distance-related theory from another perspective, that is, in addition to the significant impact of psychological distance on people’s cognition and by extension, on their behavior (Levin et al., 2002), including the impact of psychological distance on people’s moods, emotions should also receive attention. As existing research shows, in the spatial dimension, attention to environmental issues tends to be stronger at psychological distances, and our studies laterally pave the way for the intrinsic mechanisms involved, that is, the phenomenon that is most likely related to different emotions triggered by psychological distance.

Third, we explored in detail the intrinsic mechanisms by which psychological distance plays a role in eliciting emotional factors and thus influencing people’s willingness to buy green products. It has been shown that psychological distance can have a negative effect on facilitating people to solve environmental problems (McDonald et al., 2015), and we further demonstrate that emotions, especially fear, hope, and shame, play a crucial role.

## Managerial Implications

For example, if the target market is located in a region with more environmental problems or the features of the green product to be released have a greater impact on that region, the positive effects of the product and the benefits of its use should be emphasized. In regions with less prominent environmental problems, the serious consequences of ignoring the environment should be emphasized, and the possible serious impacts of continuing to ignore green products. This maximizes sales of the product.

In addition, the study also emphasizes the important role of emotions in influencing people’s green product purchasing behavior; that is, for green product manufacturers, the promotion of their products should also focus on stimulating consumer emotions. Manufacturers should explain in detail the consequences of using green products, and try to design promotional scenarios with a sense of substitution, in order to stimulate people’s resonance with the promotion of product functions, while enhancing people’s environmental awareness, and thus increasing consumers’ green purchasing behavior.

That said, we also understand that the negative emotion of shame can be very bad for people to feel, and while it drives people to compensate for the consumption of green products, we recommend that manufacturers use negative messages wisely and do not over-amplify the effects of negative emotions, such as shame in order to pursue short-term sales results.

## Limitations and Directions for Future Research

The first point is that we are only concerned with the willingness of the participants to buy green products, and we do not define in detail whether this has significantly increased their environmental awareness, which is also an indispensable factor for environmental sustainability.

Second, in addition to emotional factors, other non-emotional factors also play an important role in promoting people’s green behavior, such as the fact that different levels of detail or amounts of information that consumers may receive can also have an impact on their purchasing behavior. In this study, we tried to avoid such factors by controlling the scenario material, but we can try to observe the impact of such factors in future studies.

Third, we mainly manipulated the differences in the spatial dimensions of psychological distance, and while there are four dimensions of psychological distance itself, namely the temporal, spatial, social, and probabilistic dimensions, whether the other dimensions of psychological distance have more interesting or effective effects is also one of the directions of our further research.

Finally, it is evident from the study that sentiment has a clear impact on consumers’ willingness to buy green, and that this framing effect is not the only marketing strategy that can actually do so; many other marketing tactics (e.g., hunger marketing, public buyer reviews, etc.) may also have a significant impact on consumer sentiment, and there are a number of interesting entry points that deserve further study.

## Data Availability Statement

The raw data supporting the conclusions of this article will be made available by the authors, without undue reservation.

## Ethics Statement

Ethical review and approval was not required for the study on human participants in accordance with the local legislation and institutional requirements. Written informed consent from the participants was not required to participate in this study in accordance with the national legislation and the institutional requirements. However, written informed consent was implied via completion of the survey.

## Author Contributions

ZT, DL, FM, and XX: topic proposed. ZT and FM: experimental design and data collection. DL: manuscript writing. ZT and XX: content proofreading. All authors: contributed to the article and approved the submitted version.

## Conflict of Interest

The authors declare that the research was conducted in the absence of any commercial or financial relationships that could be construed as a potential conflict of interest.

## Appendix

### Study 1: Green Battery Ad 1

If you do not buy this new green battery, it will lead to further environmental degradation in your city, a significant increase in the safety of your water and even a serious threat to your life and health. However, this green battery is 20% more expensive than regular batteries.

### Study 2: Negative Framing Information

Our country emits approximately 50 kg of carbon per person per day, and it will take a thousand trees to fully absorb it, and global warming will cause global sea levels to rise. If everyone does not take active action to reduce the amount of carbon emissions, the consequences are unimaginable. Your city will face flooding, erosion of coastal lowlands and coasts, contamination of water in coastal areas, salinization of farmland, and even the risk of an entire city being swallowed up by the sea.

### Study 2: Positive Framing Information

Global warming will lead to a rise in sea-level, especially in the coastal cities. However, if we take timely action to adopt green and energy-saving lifestyles, the sea level rise problem will be alleviated, and will not bring great harm for a long time, and at the same time, we can also prevent the global ecology from being damaged, and also make a contribution to improving the living environment.

## References

[B1] Arthur D. Quester P. (2004). Who’s afraid of that ad? Applying segmentation to the protection motivation model. *Psychol. Mark.* 21 671–696. 10.1002/mar.20024

[B2] Ashton-James C. E. Tracy J. L. (2012). Pride and Prejudice: how Feelings about the Self Influence Judgments of Others. *Pers. Soc. Psychol. Bull.* 38 466–476. 10.1177/0146167211429449 22109249

[B3] Ausubel D. P. (1955). Relationships between shame and guilt in the socializing process. *Psychol. Rev.* 62 378–390. 10.1037/h0042534 13254977

[B4] Cabanac M. (1971). Physiological role of pleasure. *Science* 173 1103–1107. 10.1126/science.173.4002.1103 5098954

[B5] Carver C. (2003). Pleasure as a sign you can attend to something else: placing positive feelings within a general model of affect. *Cogn. Emot.* 17 241–261. 10.1080/02699930302294 29715724

[B6] Cheavens J. Feldman D. Gum A. Michael S. Snyder C. (2006). Hope therapy in a community sample: a pilot investigation. *Soc. Indic. Res.* 77, 61–78.

[B7] Dale A. (2008). Enterprise: green products gain from new price equation; they fifind new buyers as high energy costs hurt regular brands. *Wall Street Journal* Eastern Edition 251 B7

[B8] De Hooge I. E. Breugelmans S. M. Zeelenberg M. (2008). Not so ugly after all: when shame acts as a commitment device. *J. Pers. Soc. Psychol.* 95 933–943. 10.1037/a0011991 18808269

[B9] De Hooge I. E. Zeelenberg M. Breugelmans S. M. (2010). Restore and protect motivations following shame. *Cogn. Emot.* 24 111–127. 10.1080/02699930802584466

[B10] De Hooge I. E. Zeelenberg M. Breugelmans S. M. (2011). A functionalist account of shame-induced behaviour. *Cogn. Emot.* 25 939–946. 10.1080/02699931.2010.516909 21824031

[B11] De Martino B. Kumaran D. Seymour B. Dolan R. J. (2006). Frames, biases, and rational decision -making in the human brain. *Science* 313 684–687. 10.1126/science.1128356 16888142 PMC2631940

[B12] Delmas M. A. Burbano V. C. (2011). The drivers of greenwashing. *Calif. Manag. Rev.* 54 64–87. 10.1525/cmr.2011.54.1.64 33021500

[B13] Dijkstra A. Rothman A. Pietersma S. (2011). The persuasive effects of framing messages on fruit and vegetable consumption according to regulatory focus theory. *Psychol. Health* 26 1036–1048. 10.1080/08870446.2010.526715 21598188

[B14] Feldman D. B. Snyder C. R. (2005). Hope and the Meaningful Life: theoretical and empirical associations between goal–directed thinking and life meaning. *J. Soc. Clin. Psychol.* 24 401–421. 10.1521/jscp.24.3.401.65616

[B15] Han D. Duhachek A. Agrawal N. (2014). Emotions shape decisions through construal level: the case of guilt and shame. *J. Consum. Res.* 41 1047–1064. 10.1086/678300

[B16] Han S. Lerner J. S. Keltner D. (2007). Feelings and Consumer Decision Making: The Appraisal-Tendency Framework. *J. Consum. Psychol.* 17 158–168. 10.1016/s1057-7408(07)70023-2

[B17] Haws K. Winterich K. P. Naylor R. W. (2014). Seeing the world through GREEN-tinted glasses: green consumption values and responses to environmentally friendly products. *J. Consum. Psychol.* 24 336–354. 10.1016/j.jcps.2013.11.002

[B18] Hayes A. F. (2013). *Introduction to Mediation, Moderation, and Conditional Process Analysis: A Regression-Based Approach.* New York: Guilford Press.

[B19] Higgins E. T. (1987). Self-discrepancy: a theory relating self and affect. *Psychol. Rev.* 94 319–340. 10.1037/0033-295x.94.3.319 3615707

[B20] Hoelter J. W. (1979). Multidimensional treatment of fear of death. *J. Consult. Clin. Psychol.* 47 996–999. 10.1037/0022-006x.47.5.996 512155

[B21] Kalamas M. Cleveland M. Laroche M. (2014). Pro-environmental behaviors for thee but not for me: green giants, green Gods, and external environmental locus of control. *J. Bus. Res.* 67 12–22. 10.1016/j.jbusres.2013.03.007

[B22] Kemper T. D. (1978). *A Social Interactional Theory of Emotions.* New York, NY: Wiley.

[B23] Leiserowitz A. Maibach E. Roser-Renouf C. Feinberg G. Howe P. (2013). *Climate Change in the American Mind: Americans’ Global Warming Beliefs and Attitudes in April 2013.* New Haven, CT: Yale University and George Mason University.

[B24] Levin I. P. Gaetha G. J. Schreiber J. Lauriola M. (2002). A new look at framing effects: distribution of effect sizes, individual differences, and independence of types of effects. *Organ. Behav. Hum. Decis. Process.* 88 411–429. 10.1006/obhd.2001.2983

[B25] Levin I. P. Schneider S. L. Gaeth G. J. (1998). All frames are not created equal: a typology and critical analysis of framing effects. *Organ. Behav. Hum. Decis. Process.* 76 149–188. 10.1006/obhd.1998.2804 9831520

[B26] Lin Y. C. Chang C. C. A. (2012). Double standard: the role of environmental consciousness in green product usage. *J. Mark.* 76 125–134. 10.1509/jm.11.0264 11670861

[B27] Liu Z. S. Gu D. (2020). Gain or Loss? The effect of goal framing on green consumption intention.Chinese. *J. Clin. Psychol.* 28:99.

[B28] Loroz P. S. (2007). The interaction of message frames and reference points in prosocial persuasive appeals. *Psychol. Mark.* 24 1001–1023. 10.1002/mar.20193

[B29] McDonald R. I. Chai H. Y. Newell B. R. (2015). Personal experience and the ‘psychological distance’ of climate change: an integrative review. *J. Environ. Psychol.* 44 109–118. 10.1016/j.jenvp.2015.10.003

[B30] McElroy T. Seta J. J. (2004). On the other hand am I rational? Hemispheric activation and the framing effect. *Brain Cogn.* 55 572–580. 10.1016/j.bandc.2004.04.002 15223204

[B31] Moons I. De Pelsmacker P. (2012). Emotions as determinants of electric car usage intention. *J. Mark. Manage.* 28 195–237. 10.1080/0267257x.2012.659007

[B32] Niedenthal P. M. Tangney J. P. Gavanski I. (1994). “If only I weren’t” versus “If only I hadn’t”: distinguishing shame and guilt in counterfactual thinking. *J. Pers. Soc. Psychol.* 67 585–595. 10.1037/0022-3514.67.4.585 7965606

[B33] Olsen M. C. Slotegraaf R. J. Chandukala S. R. (2014). Green claims and message frames: how green new products change brand attitude. *J. Mark.* 78 119–137. 10.1509/jm.13.0387 11670861

[B34] O’Neill S. Nicholson-Cole S. (2009). Fear Won’t Do It. *Sci. Commun.* 30 355–379.

[B35] Patrick V. M. Chun H. E. MacInnis D. J. (2009). Affective forecasting and self-control: when anticipating pride wins over anticipating shame in a self-regulation context. *J. Consum. Psychol.* 19 537–545. 10.1016/j.jcps.2009.05.006

[B36] Pekrun R. (2006). The control-value theory of achievement emotions: assumptions, corollaries, and implications for educational research and practice. *Educ. Psychol. Rev.* 18 315–341. 10.1007/s10648-006-9029-9

[B37] Romani S. Grappi S. Bagozzi R. P. (2016). Corporate socially responsible initiatives and their effects on consumption of green products. *J. Bus. Ethics* 135 253–264. 10.1007/s10551-014-2485-0

[B38] Roseman I. J. (1991). Appraisal determinants of discrete emotions. *Cogn. Emot.* 3 161–200. 10.1080/02699939108411034

[B39] Roseman I. J. Wiest C. Swartz T. S. (1994). Phenomenology, behaviors, and goals differentiate discrete emotions. *J. Pers. Soc. Psychol.* 2 206–221. 10.1037/0022-3514.67.2.206

[B40] Ruiter R. A. C. Abraham C. Kok G. (2001). Scary warnings and rational precautions: a review of the psychology of fear appeals. *Psychol. Health* 16 613–630. 10.1080/08870440108405863

[B41] She S. Tian Y. Lu L. Eimontaite I. Xie T. Sun Y. (2019). An exploration of hiking risk perception: dimensions and antecedent factors. *Int. J. Environ. Res. Public Health* 16:1986. 10.3390/ijerph16111986 31167460 PMC6603918

[B42] Snyder C. R. (1995). Conceptualizing, measuring, and nurturing hope. *J. Couns. Dev.* 73 355–360. 10.1002/j.1556-6676.1995.tb01764.x

[B43] Sun Y. Li P. She S. Eimontaite I. Yang B. (2018). Boosting water conservation by improving campaign: evidence from a field study in China. *Urban Water J.* 15 966–973. 10.1080/1573062x.2019.1581233

[B44] Sun Y. Li Y. Cai B.-F. Li Q. (2020). ‘Comparing the explicit and implicit attitudes of energy stakeholders and the public towards carbon capture and storage’. *J. Clean. Prod.* 254:120051 10.1016/j.jclepro.2020.120051

[B45] Tangney J. P. Dearing R. L. (2002). *Shame and Guilt (Emotions and Social Behavior).* New York, NY: Guildford.

[B46] Tracy J. L. Robins R. W. (2006). Appraisal antecedents of shame and guilt: support for a theoretical model. *Pers. Soc. Psychol. Bull.* 36 3–4. 10.1177/0146167206290212 16963605

[B47] Tversky A. Kahneman D. (1981). The framing of decisions and the psychology of choice. *Science* 211 453–458. 10.1126/science.7455683 7455683

[B48] Van Doorn J. Verhoef P. C. (2011). Willingness to pay for organic products: differences between virtue and vice foods. *Int. J. Res. Mark.* 28 167–180. 10.1016/j.ijresmar.2011.02.005

[B49] Watson D. Clark L. A. Tellegen A. (1988). Development and validation of brief measures of positive and negative affect: the PANAS scales. *J. Pers. Soc. Psychol.* 54 1063–1070. 10.1037/0022-3514.54.6.1063 3397865

[B50] Weiner B. (1985). An attributional theory of achievement motivation and emotion. *Psychol. Rev.* 92:548 10.1037/0033-295x.92.4.548 3903815

[B51] White K. MacDonnell R. Dahl D. W. (2011). It’s the mindset that matters: the role of construal level and message framing in influencing consumer efficacy and conservation behaviors over the long-term. *Journal of Mark. Res.* 48 472–485. 10.1509/jmkr.48.3.472 11670861

[B52] Wilcox K. Kramer T. Sen S. (2010). Indulgence or self-control: a dual process model of the effect of incidental pride on indulgent choice. *J. Consum. Res.* 38 151–163. 10.1086/657606

[B53] Williams L. A. DeSteno D. (2009). Pride: adaptive social emotion or seventh sin? *Psychol. Sci.* 20 284–288. 10.1111/j.1467-9280.2009.02292.x 19207690

[B54] Witte K. (1992). Putting the fear back into fear appeals: the extended parallel process model. *Commun. Monogr.* 59 329–349. 10.1080/03637759209376276

